# SIRT1 Modulates the Photodynamic Anticancer Activity of 5,10,15-Triethoxycarbonyl P(V) Corrole in Hepatocellular Carcinoma

**DOI:** 10.3390/ph18081226

**Published:** 2025-08-20

**Authors:** Yan Liu, Jian Zheng, Jiayi Zhu, Xuemin Xian, Zhao Zhang, Haitao Zhang

**Affiliations:** 1 Department of Biochemistry and Molecular Biology, School of Basic Medicine, Guangdong Medical University, Zhanjiang 524023, China; liuy598@mail2.sysu.edu.cn (Y.L.); 18816799683@163.com (J.Z.); xueminxian@gdmu.edu.cn (X.X.); 2 Hubei Key Laboratory of Pollutant Analysis & Reuse Technology, College of Chemistry and Chemical Engineering, Hubei Normal University, Huangshi 435002, China; zhujy_89@163.com

**Keywords:** corrole, photodynamic therapy, hepatocellular carcinoma, SIRT1, apoptosis

## Abstract

**Background**: Hepatocellular carcinoma (HCC) remains a global health challenge with limited therapeutic efficacy. Photodynamic therapy (PDT) using 5,10,15-triethoxycarbonyl P(V) corrole (**1-P**) shows promise, but its molecular mechanisms and regulatory factors, particularly the role of SIRT1, are poorly understood. **Methods**: The effects of **1-P** combined with red light irradiation (625 nm) on HCC cells (HepG2, PLC/PRF5, MHCC97H) were evaluated via MTT, clonogenic assays, flow cytometry (apoptosis, mitochondrial membrane potential, ROS), and Western blotting (p53, Bax, Bcl-2, cleaved caspase-3, SIRT1). SIRT1-overexpressing cells and xenograft mouse models were used to validate its regulatory role. **Results**: **1-P** with irradiation dose-dependently inhibited cell viability (IC50: 0.965–1.478 μM), suppressed clonogenicity, induced apoptosis (up to 68.8%), reduced mitochondrial membrane potential, and elevated ROS. Mechanistically, **1-P** upregulated Bax/p53/cleaved caspase-3 and downregulated Bcl-2/SIRT1. SIRT1 overexpression rescued **1-P**-induced apoptosis (30–50% reduction), restored mitochondrial function, and attenuated ROS accumulation. In vivo, **1-P** significantly inhibited tumor growth in mice, but SIRT1 overexpression diminished this effect (*p* < 0.05). **Conclusions**: **1-P** exerts potent photodynamic anticancer effects via mitochondrial dysfunction, oxidative stress, and apoptosis induction. SIRT1 is a critical modulator of **1-P** activity, highlighting its potential as a therapeutic target to enhance PDT efficacy in HCC.

## 1. Introduction

Cancer remains one of the most challenging diseases to treat globally, with hepatocellular carcinoma (HCC) being a leading cause of cancer-related mortality worldwide [1]. In the United States, HCC ranks as the fifth deadliest cancer and is unique among the top five due to its rising incidence [2]. In China, HCC holds the fourth-highest rate of new cases and the second-highest mortality rate [3]. Current therapeutic strategies, such as chemotherapy and immunotherapy, primarily target tumor cell proliferation or induce cytotoxicity. However, their efficacy is limited by systemic toxicity and the development of drug resistance [4,5]. Consequently, enhancing tumor cell sensitivity to therapeutic interventions has emerged as a critical focus in oncology research.

Photodynamic therapy (PDT), a clinically established anticancer approach for over four decades, utilizes photosensitizers (PS) that selectively accumulate in malignant tissues [6]. Upon irradiation with light at specific wavelengths matching the PS absorption peak, reactive oxygen species (ROS) are generated, triggering localized tumor cell death [7]. Porphyrin derivatives, widely used as PS agents, have demonstrated significant clinical success. Corrole—a synthetic porphyrin analog sharing structural homology with vitamin B12 (corrin)—exhibits superior properties, including lower molecular weight, higher fluorescence quantum yield, and enhanced photostability compared to traditional porphyrins [8,9]. Recent studies highlight the potential of 5,10,15-Triethoxycarbonyl P(V) Corrole (**1-P**) as a tumor-targeting PS. Preclinical data reveal that **1-P** exhibits high efficacy and low toxicity, with a safe dose exceeding 20 mg/kg in mice, far above its therapeutic PDT dose (1 mg/kg), underscoring its translational promise [10].

SIRT1, a NAD^+^-dependent deacetylase, regulates apoptosis by modulating acetylation levels of proteins such as p53 and FOXO1. Previous studies demonstrate that SIRT1 deacetylates p53 to suppress apoptosis, whereas cisplatin-induced SIRT1 inactivation promotes p53 acetylation and apoptosis [11,12]. Our group previously observed that **1-P**-mediated PDT significantly reduces SIRT1 protein levels in HCC cells [10]. However, the mechanistic role of SIRT1 in **1-P**’s anticancer activity remains unexplored. Herein, we investigated the effects of **1-P** combined with red light (625 nm) on HCC cell lines (HepG2, PLC/PRF5, MHCC97H) through viability assays, mitochondrial function analysis, ROS quantification, and in vivo xenograft models. Our findings elucidate SIRT1’s regulatory role in **1-P**-driven photodynamic activity, providing novel insights into corrole-based PDT mechanisms.

## 2. Results

### 2.1. Synergistic Cytotoxicity of 1-P Combined with Red Light Irradiation in Hepatocellular Carcinoma Cells

**Figure 1:**
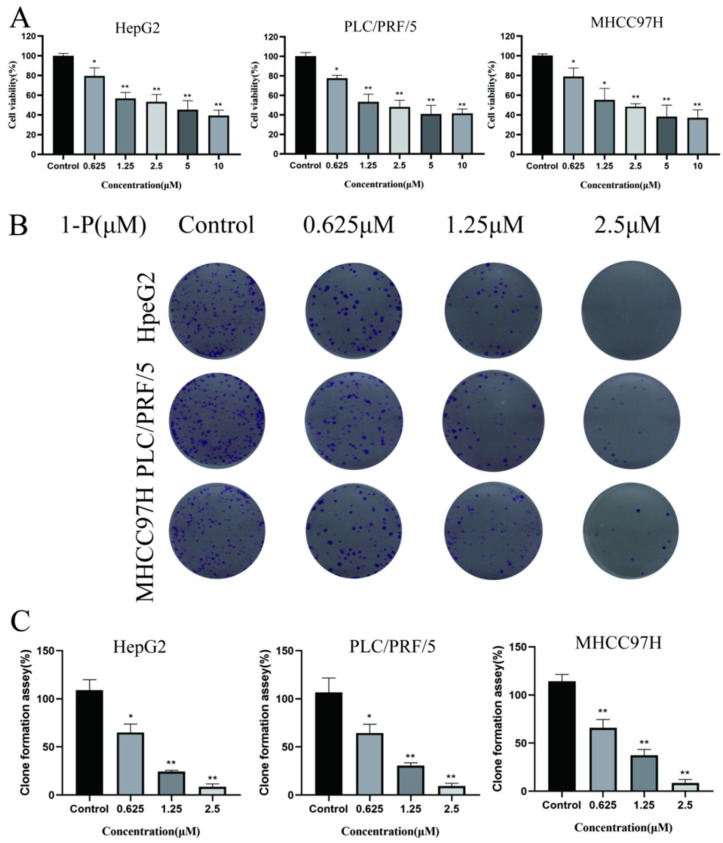
**1-P** combined with red light irradiation exerts cytotoxic effects on hepatocellular carcinoma cells. (**A**) Cell viability of HepG2, PLC/PRF/5, and MHCC97H cells treated with **1-P** (0.625–10 μM) for 4 h, followed by red light irradiation (625 nm, 5 W, 1 h) and 20 h incubation. (**B**) Representative images (1×) and (**C**) quantification of clonogenic survival in cells treated with **1-P** (0.625–2.5 μM) and red light. Data are mean ± SD; * p < 0.05, ** p < 0.01 vs. control.

HepG2, PLC/PRF/5, and MHCC97H cells were treated with varying concentrations of **1-P** (10, 5, 2.5, 1.25, 0.625 μM) for 4 h, followed by 24 h incubation in the dark. MTT assays showed no significant differences in cell viability between **1-P-**treated groups and controls (Figure S1), indicating no intrinsic cytotoxicity of **1-P** alone. However, when cells were irradiated with red light (625 nm, 5 W, 1 h) after 4 h of **1-P** treatment and further incubated for 20 h, a significant reduction in cell viability was observed (Figure 1A). IC50 values under light co-treatment were calculated as 0.965 μM (HepG2), 1.202 μM (PLC/PRF/5), and 1.478 μM (MHCC97H). Based on these results, 2.5, 1.25, and 0.625 μM **1-P** were selected for subsequent experiments, all combined with red light irradiation. Clonogenic assays demonstrated a concentration-dependent decrease in colony formation across all cell lines compared to controls (Figure 1B,C), confirming that **1-P** synergizes with red light to suppress clonogenic survival in hepatocellular carcinoma cells.

### 2.2. 1-P Induces Concentration-Dependent Apoptosis in Hepatocellular Carcinoma Cells via Modulation of SIRT1, Bcl-2, and p53 Signaling Pathways

**Figure 2:**
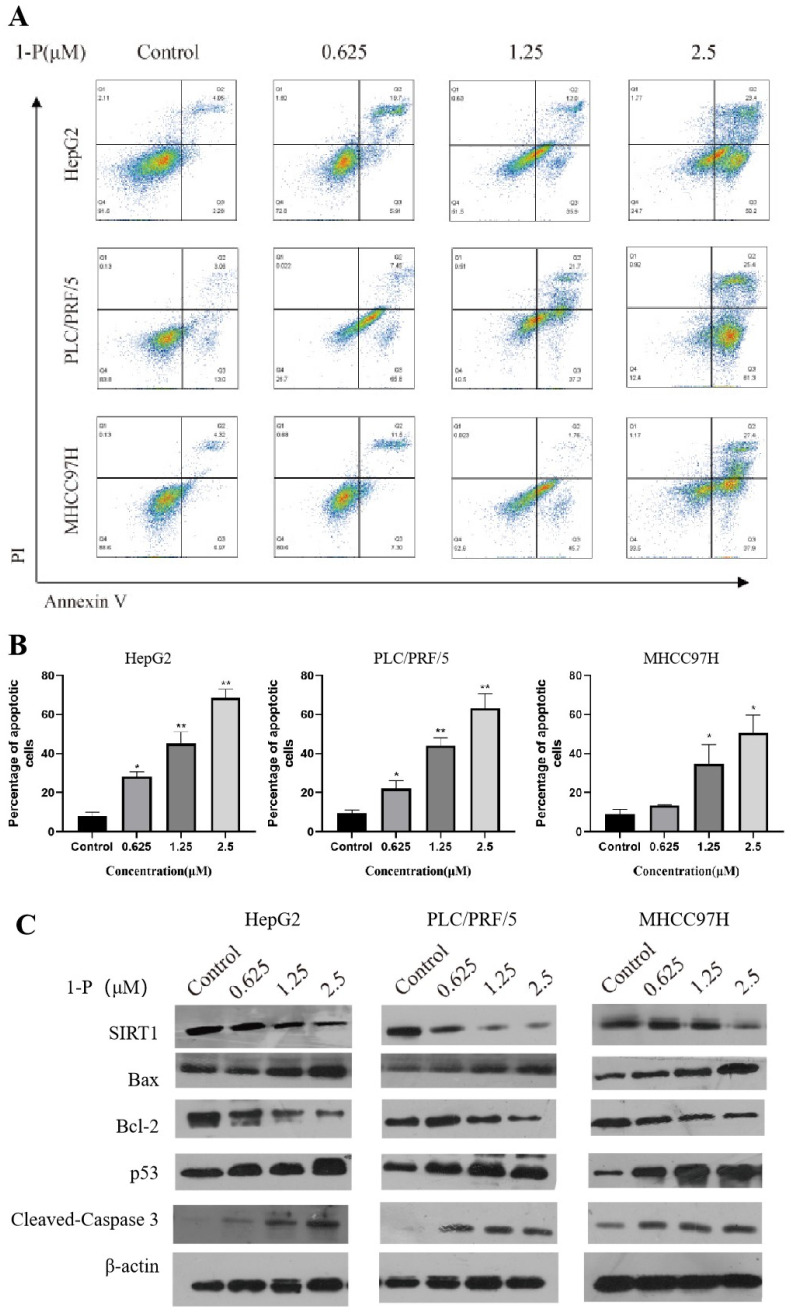
**1-P** induces apoptosis in hepatocellular carcinoma cells via the regulation of apoptosis-related proteins. (**A**,**B**) Flow cytometry analysis of apoptosis in HepG2, PLC/PRF/5, and MHCC97H cells treated with **1-P** (0.625, 1.25, 2.5 μM) followed by 625 nm light irradiation (1 h). Each point in the figure represents a cellular granule, with colors ranging from blue to red indicating the cell count from low to high. * represents *p* < 0.05 and ** represents *p* < 0.01. (**C**) Western blot showing expression levels of apoptosis-related proteins (Bcl-2, SIRT1, BAX, p53, cleaved caspase-3) and β-actin (loading control).

HepG2, PLC/PRF/5, and MHCC97H cells were treated with varying concentrations of **1-P** (2.5, 1.25, 0.625 μM). After 4 h of drug administration, all groups were irradiated with 625 nm light for 1 h, followed by 20 h of incubation. Compared to the control group, **1-P** treatment significantly increased apoptosis rates in a concentration-dependent manner (Figure 2A,B). Specifically, apoptosis rates in HepG2 cells reached 28.24%, 44.27%, and 68.80% at 0.625, 1.25, and 2.5 μM **1-P**, respectively. Similarly, PLC/PRF/5 cells exhibited apoptosis rates of 20.04%, 44.97%, and 67.02%, while MHCC97H cells showed rates of 13.51%, 34.66%, and 50.02% at the respective concentrations. These results indicate that **1-P** effectively induces apoptosis in all three hepatocellular carcinoma cell lines, with the most pronounced effect observed in HepG2 cells. Following the same treatment protocol (2.5, 1.25, 0.625 μM **1-P** with 625 nm irradiation), Western blot analysis revealed that **1-P** significantly reduced Bcl-2 expression while upregulating SIRT1, BAX, p53, and cleaved caspase-3 levels in HepG2, PLC/PRF/5, and MHCC97H cells compared to controls (Figure 2C). These findings demonstrate that **1-P** promotes apoptosis in hepatocellular carcinoma cells by modulating key regulatory proteins associated with apoptotic pathways.

### 2.3. 1-P Induces Mitochondrial Dysfunction and ROS Accumulation to Promote Apoptosis in Hepatocellular Carcinoma Cells

HepG2, PLC/PRF/5, and MHCC97H cells were treated with **1-P** (0.625, 1.25, 2.5 μM) for 4 h, followed by 625 nm light irradiation (1 h) and 20 h of incubation. Mitochondrial membrane potential (ΔΨm) was assessed using JC-1 staining. Compared to controls, **1-P** treatment induced a concentration-dependent decrease in red/green fluorescence ratio (indicating ΔΨm collapse) across all cell lines (Figure S2A,B), suggesting mitochondrial apoptosis pathway activation. Concurrently, intracellular reactive oxygen species (ROS) levels were significantly elevated in **1-P-**treated groups (Figure S2C,D), with fluorescence intensity increasing by 1.5- to 3.2-fold compared to untreated controls. These results demonstrate that **1-P** triggers mitochondrial dysfunction and oxidative stress, collectively contributing to apoptosis in hepatocellular carcinoma cells.

### 2.4. SIRT1 Overexpression Attenuates 1-P-Induced Hepatocellular Carcinoma Cell Apoptosis via Mitochondrial Protection and Oxidative Stress Suppression

HepG2, PLC/PRF/5, and MHCC97H hepatocellular carcinoma cells were transiently transfected with SIRT1-overexpressing plasmids, treated with 1.25 μM **1-P** for 4 h, exposed to 625 nm light irradiation (1 h), and further incubated for 20 h. MTT assays revealed that SIRT1 overexpression had no baseline effect on cell viability but significantly rescued **1-P-**induced cytotoxicity (Figure S3A). Furthermore, colony formation assays demonstrated that SIRT1 overexpression did not alter the basal clonogenic ability, yet it markedly restored the **1-P-**impaired colony-forming capacity (Figure S3B,C).

**Figure 3:**
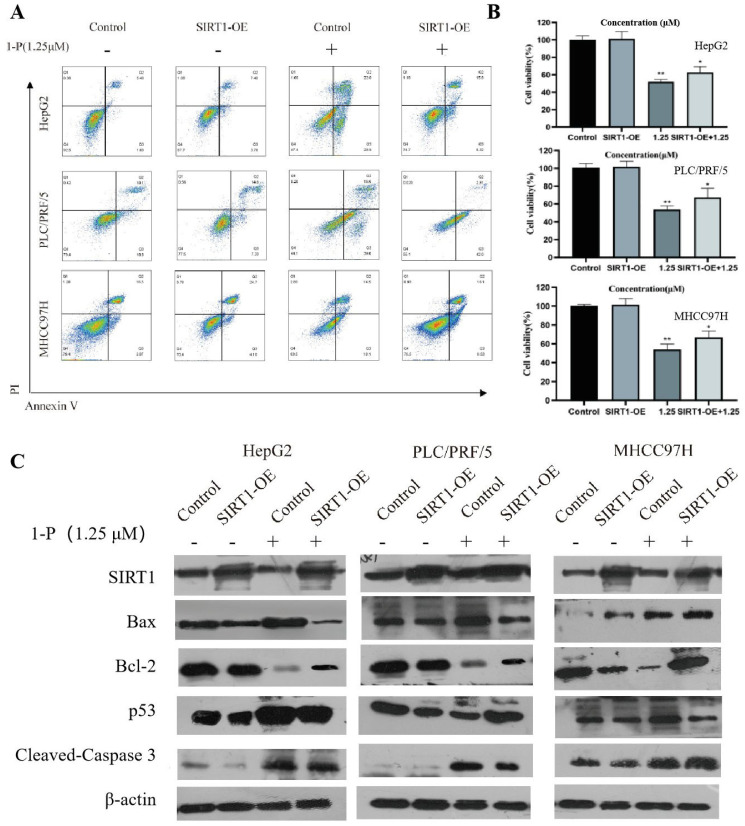
SIRT1 overexpression attenuates **1-P**-induced apoptosis in hepatocellular carcinoma cells. (**A**,**B**) Representative flow cytometry plots (Annexin V/PI staining) of HCC cells transiently transfected with SIRT1-overexpressing plasmids (SIRT1-OE) and treated with 1.25 μM **1-P** (4 h with 625 nm light irradiation). Each point in the figure represents a cellular granule, with colors ranging from blue to red indicating the cell count from low to high. * represents *p* < 0.05 and ** represents *p* < 0.01. (**C**) Western blot showing expression levels of apoptosis-related proteins (Bcl-2, SIRT1, BAX, p53, cleaved caspase-3) and β-actin (loading control).

Transient overexpression of SIRT1 (SIRT1-OE) in HepG2, PLC/PRF/5, and MHCC97H cells followed by 1.25 μM **1-P** treatment (4 h with 625 nm light irradiation) significantly attenuated **1-P-**induced apoptosis, with apoptosis rates decreasing by 30–50% compared to the **1-P-**treated group, while showing no baseline effect versus untreated controls (flow cytometry, Figure 3A,B). Western blot analysis revealed that SIRT1 overexpression upregulated anti-apoptotic Bcl-2 expression and suppressed pro-apoptotic markers, including BAX, p53, and cleaved caspase-3 (Figure 3C), indicating SIRT1-mediated regulation of apoptotic pathways counteracts **1-P-**induced cell death.

SIRT1-overexpressing (SIRT1-OE) HepG2, PLC/PRF/5, and MHCC97H cells were treated with 1.25 μM **1-P** (4 h with 625 nm light irradiation) and analyzed after 20 h. JC-1 staining revealed that SIRT1 overexpression restored mitochondrial membrane potential (ΔΨm) in **1-P-**treated cells, as evidenced by increased red fluorescence and reduced green fluorescence intensity, while showing no effect on untreated controls (Figure S4A,B). Furthermore, DCFH-DA assays demonstrated that SIRT1 overexpression reduced **1-P-**induced reactive oxygen species (ROS) accumulation by 40–60% compared to the **1-P-**treated group, without altering baseline ROS levels (Figure S4C,D). These findings indicate that SIRT1 mitigates **1-P-**induced cytotoxicity through dual mechanisms of mitochondrial protection and oxidative stress suppression.

### 2.5. Anti-Tumor Effects of 1-P in HepG2 and SIRT1-Stably Overexpressing HepG2 Tumor-Bearing Mice

**Figure 4:**
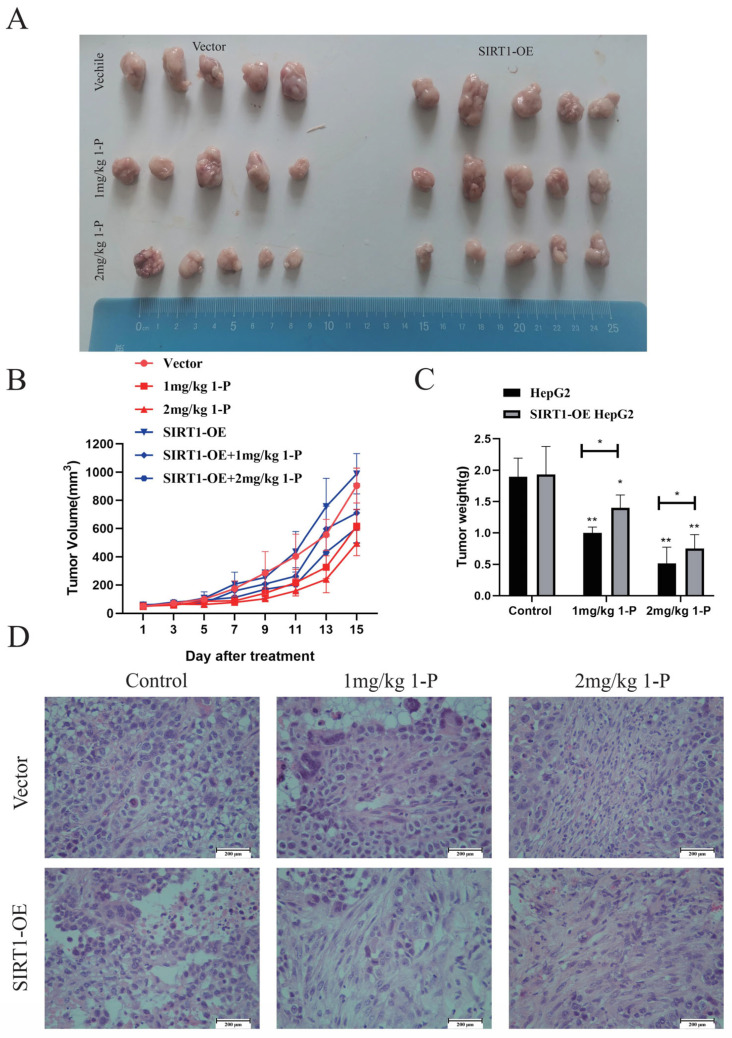
**1-P** suppresses tumor growth in a SIRT1-dependent manner. (**A**) Representative tumor images from nude mice bearing HepG2 xenografts (Vector: empty lentivirus control; SIRT1-OE: SIRT1-overexpressing). (**B**) Tumor growth kinetics showing volume reduction in **1-P**-treated groups (*n* = 5, mean ± SD). (**C**) Terminal tumor weights demonstrating enhanced **1-P** efficacy in Vector controls versus SIRT1-OE group (* represents *p* < 0.05 and ** represents *p* < 0.01). (**D**) H&E-stained tumor sections (100×) revealing reduced necrosis areas in **1-P**-treated Vector controls.

Lentivirus-mediated SIRT1-stably overexpressing HepG2 cells (Figure S5) were subcutaneously implanted into mice for **1-P** treatment. Both HepG2 and SIRT1-overexpressing (SIRT1-OE) HepG2 groups showed divergent tumor volumes from the control group starting at day 11. Post-treatment analysis revealed concentration-dependent tumor weight reduction in both groups, though SIRT1-OE tumors exhibited significantly higher weights than wild-type HepG2 tumors (* p < 0.05), with reduced necrotic areas, indicating SIRT1 overexpression attenuated the tumor-suppressive effects of **1-P** (Figure 4). No significant body weight changes or histopathological abnormalities in heart, liver, spleen, lung, or kidney tissues were observed (Figure S6), confirming **1-P**’s favorable safety profile.

## 3. Discussion

PDT has emerged as a prominent research direction in tumor treatment. Essentially, PDT involves the use of tumor-localized photosensitizers activated by specific light sources, stimulating the generation of ROS. The resulting ROS can damage cellular macromolecules, leading to tumor cell death. Sirtuin 1 (SIRT1), a nicotinamide adenine dinucleotide (NAD^+^)-dependent deacetylase, is the most well-characterized member of the sirtuin family. SIRT1 is widely expressed in various somatic cells, primarily localized in the nucleus. Its regulatory roles in energy metabolism, oxidative stress, tumorigenesis, and aging largely depend on its deacetylase activity. By modulating the acetylation levels of histones, transcription factors, and signaling molecules, SIRT1 influences gene transcription [13,14,15]. This study investigates the mechanism by which a metal-corrole complex (**1-P**) inhibits HCC cell growth.

Our findings indicate that **1-P** alone does not significantly affect HCC cell viability, but when combined with red light irradiation, it reduces cell activity. This suggests that **1-P** requires photoactivation to exert its pharmacological effects. Further experiments demonstrated that **1-P** suppresses clonogenic ability, induces apoptosis, alters protein expression, decreases mitochondrial membrane potential (ΔΨm), and elevates intracellular ROS levels. These effects may collectively contribute to its antitumor activity. Previous studies support these observations. Zhang et al. reported that a gallium(III) tri(ethoxy-carbonyl)corrole induced A549 cell apoptosis via ROS-mediated mitochondrial dysfunction [16,17]. Similarly, Sun et al. demonstrated that hydroxyl-corrole and its gallium(III) complex exhibited phototoxicity against MDA-MB-231 and 4T1 cells upon 625 nm irradiation, arresting cells in the G1 phase through ROS generation [18]. Our results suggest that **1-P** may promote HCC cell apoptosis by upregulating pro-apoptotic proteins, impairing mitochondrial function, and disrupting cell cycle progression.

We observed that **1-P** increases ROS levels in HCC cells post-irradiation, potentially inducing oxidative stress and cellular damage. Oxidative stress arises from an imbalance between ROS production and antioxidant defenses, leading to DNA and protein damage. Cen et al. found that a porphyrin-derived compound induced ROS-mediated oxidative damage in HepG2 and HeLa cells, triggering apoptosis [19]. Soll et al. reported that a lipophilic corrole induced necrosis via ROS-dependent plasma membrane destabilization and lysosomal stress [20]. Zhang et al. further demonstrated a water-soluble corrole localized in cytoplasmic organelles, generating ROS that caused ΔΨm collapse and DNA damage [21]. Oxidative stress is implicated in various degenerative diseases, including cancer. ROS-mediated protein modifications can impair cellular function, and mitochondrial permeability changes may release pro-apoptotic factors. Our data suggest that **1-P** exerts its antitumor effects primarily through ROS generation.

To explore SIRT1’s role, we overexpressed SIRT1 in HCC cells and treated them with **1-P** (1.25 μM) followed by 625 nm irradiation. Compared to the **1-P**-only group, SIRT1 overexpression increased HCC cell viability, restored clonogenic capacity, reduced apoptosis, modulated apoptotic protein expression, rescued ΔΨm decline, and mitigated cell cycle perturbations. These findings suggest that SIRT1 overexpression alters cellular physiology, potentially counteracting **1-P**’s antitumor effects. Zhang et al. reported that a gallium(III) corrole reduced SIRT1 expression in A549 cells, enhancing apoptosis. Similarly, their cationic sulfonated corrole decreased SIRT1 levels upon irradiation [16]. These results underscore SIRT1’s regulatory role in **1-P**-mediated HCC cell inhibition.

We evaluated **1-P**’s efficacy in HepG2 xenograft mice, including a SIRT1-overexpressing HepG2 model. Tumor volume differences between control and treatment groups emerged by day 11. Post-treatment, tumor weight decreased dose-dependently, with significant differences between wild-type and SIRT1-overexpressing tumors. Histological analysis revealed reduced necrosis, confirming **1-P**’s tumor-suppressive effects. Notably, SIRT1 overexpression attenuated **1-P**’s efficacy, highlighting its role in treatment resistance. No significant weight changes or organ toxicity were observed in treated mice, indicating **1-P**’s favorable safety profile. SIRT1 overexpression did not affect baseline cell viability, possibly due to functional redundancy or cell type-specific SIRT1 roles. However, the current methodology we employed involves establishing a subcutaneous tumor model in mice using cells with plasmid-mediated SIRT1 overexpression, followed by observation of subsequent tumor growth and mouse physiological/biochemical indicators. After transplantation, the tumor cells reside within a living physiological environment, making it inherently difficult to guarantee consistent expression levels throughout the entire experiment [22,23].

**1-P** exhibits potent antitumor activity against HCC via ROS generation, apoptosis induction, and mitochondrial dysfunction. SIRT1 overexpression mitigates these effects, suggesting its involvement in treatment resistance. These findings provide insights into **1-P**’s mechanism and support further development of corrole-based therapies for liver cancer.

## 4. Materials and Methods

### 4.1. Materials

Reagents and Chemicals DMEM medium, fetal bovine serum (FBS), Opti-MEM™ medium, Lipofectamine 3000 reagent, and puromycin were obtained from Gibco (Thermo Fisher Scientific, Waltham, MA, USA). Penicillin-Streptomycin (100×), 0.25% Trypsin-EDTA solution, MTT reagent, Annexin V-FITC Apoptosis Detection Kit, and Reactive Oxygen Species (ROS) Detection Kit were purchased from Beyotime Biotechnology (Shanghai, China). SIRT1-overexpressing lentivirus was provided by Jingjie Biotechnology (Hangzhou, China). PMSF, RIPA lysis buffer, and dimethyl sulfoxide (DMSO) were acquired from Qiyun Biotechnology (Guangzhou, China). Ultra-sensitive chemiluminescent substrate ECL and 5× SDS loading buffer were sourced from Dingjie Biotechnology (Zhanjiang, China). BCA Protein Assay Kit was supplied by Dingguo Biotechnology (Shanghai, China). Rabbit-derived primary antibodies against Bcl-2, SIRT1, Bax, β-actin, and cleaved caspase-3 were purchased from Cell Signaling Technology (Danvers, MA, USA). Spectra Multicolor High Range Protein Ladder was procured from Jietewei Biotechnology (Guangzhou, China). Nitrocellulose (NC) membranes were obtained from Mingzhi Biotechnology (Guangzhou, China). Phosphorus(V) tris(ethoxycarbonyl)corrole (**1-P**) was synthesized as previously described [10]. All other reagents and solvents were commercially available and used as received unless otherwise specified.

Instruments: Cell culture: HERAcell 150i CO_2_ incubator (Thermo Fisher Scientific, USA). Flow cytometry: BD FACSCanto II flow cytometer (BD Biosciences, Franklin Lakes, NJ, USA). Microplate reading: BioTek Synergy H1 microplate reader (BioTek Instruments, Winooski, VT, USA). Cell counting: Countstar BioTech automated cell counter (Ruiyu Biotechnology, Shanghai, China). Microscopy: Nikon Eclipse Ts2R fluorescence microscope and Nikon ECLIPSE Ti2 inverted fluorescence microscope (Nikon, Minato City, Japan). Centrifugation: Eppendorf 5424R refrigerated centrifuge (Eppendorf, Hamburg, Germany). Electrophoresis: Bio-Rad Mini-PROTEAN Tetra electrophoresis system (Bio-Rad, Hercules, CA, USA).

### 4.2. Cell Experiments In Vitro

#### 4.2.1. Cell Lines and Culture

Human hepatoma cell lines (HepG2, PLC/PRF/5, and MHCC97H) were provided by ATCC (Manassas, VA, USA). Cells were maintained at 37 °C in 5% CO_2_ and cultured in T75 flasks filled with 10 mL DMEM medium. The medium was supplemented with 10% heat-activated FBS and 1% Penicillin-Streptomycin. Following this, cells were harvested by 0.25% Trypsin-EDTA, resuspended in complete medium, then counted through a Leica DM6000 B optical microscope (Leica Microsystems, Wetzlar, Germany) using a standard Beckman Coulter Z2 hemocytometer (Beckman Coulter, Brea, CA, USA) before plating. Cells used in all experiments were below 5 passages.

#### 4.2.2. MTT Cell Viability Assay

Cells in the logarithmic growth phase were seeded into 96-well plates at a density of 5 × 10^3^ cells/well. After 24 h of culture, the medium was discarded, and cells were treated with 0, 0.625, 1.25, 2.5, 5, or 10 μmol/L **1-P** solution (prepared in complete DMEM medium), while the control group received complete DMEM medium alone. Following red light irradiation (625 nm, 5 W, 1 h, 15 cm distance from the light source), cells were further incubated for 23 h. Subsequently, 10 μL of 5 mg/mL MTT solution was added to each well, and plates were incubated for 4 h. The supernatant was then carefully removed, and 150 μL of DMSO was added to dissolve the formazan crystals. Plates were shaken on a microplate shaker for 15 min, and absorbance was measured at 490 nm using a microplate reader.

#### 4.2.3. Clonogenic Assay

Cells in the logarithmic growth phase were seeded into 6-well plates at a density of 1 × 10^2^ cells/well. After 24 h of culture, the medium was discarded, and cells were treated with 0, 0.625, 1.25, or 2.5 μM **1-P** solution (prepared in complete DMEM medium), while the control group received complete DMEM medium alone. Following red light irradiation (625 nm, 5 W, 1 h, 15 cm distance from the light source), cells were cultured for an additional 10 days. Cell colony morphology was observed under a microscope. The supernatant was removed, and colonies were fixed with 1 mL of 4% paraformaldehyde for 15 min, washed, and stained with 1 mL crystal violet for 15 min. After drying, colonies were photographed and counted.

#### 4.2.4. Annexin V/PI Apoptosis Assay

Cells were seeded into 6-well plates at a density of 5 × 10^5^ cells/well. After treatment as described in Section 4.2.3, cells were collected by centrifugation. According to the manufacturer’s protocol of the Annexin V-FITC/PI Apoptosis Detection Kit (BD Biosciences), harvested cells were resuspended in 500 μL 1× binding buffer, stained with 5 μL Annexin V-FITC and 5 μL PI, and incubated at room temperature in the dark for 10 min. Apoptosis rates were analyzed by flow cytometry (BD FACSCanto II).

#### 4.2.5. JC-1 Mitochondrial Membrane Potential Assay

Cells were seeded into 6-well plates at 5 × 10^5^ cells/well. After treatment as in Section 4.2.3, cells were collected by centrifugation. The JC-1 probe (Beyotime) was prepared according to the manufacturer’s instructions. Cells were incubated with 500 μL JC-1 working solution at 37 °C for 20 min, washed twice with ice-cold PBS, resuspended in 500 μL PBS, and analyzed by flow cytometry.

#### 4.2.6. DCFH-DA ROS Detection

Cells were seeded into 6-well plates at 5 × 10^5^ cells/well. After treatment as in Section 4.2.5, cells were collected by centrifugation. DCFH-DA (Sigma-Aldrich, St. Louis, MO, USA) was diluted 1:1000 in serum-free DMEM, and 500 μL of the probe solution was added to each well. Cells were incubated in the dark for 20 min (gently inverted every 5 min), washed three times with serum-free DMEM to remove the residual probe, resuspended in 500 μL PBS, and analyzed by flow cytometry.

#### 4.2.7. Western Blot Analysis

Cells were cultured in 10-cm dishes until reaching logarithmic growth. After treatment as in Section 4.2.5, cells were lysed on ice with RIPA buffer (800 μL RIPA lysis buffer, 100 μL 10× protease inhibitor cocktail, 100 μL 10× phosphatase inhibitor, and 10 μL PMSF). Lysates were scraped, centrifuged at 12,000× *g* for 10 min at 4 °C, and quantified using the BCA Protein Assay Kit (Thermo Fisher). Equal amounts of protein were mixed with the loading buffer, denatured at 100 °C for 5 min, separated by SDS-PAGE, and transferred to PVDF membranes. Membranes were blocked with 5% skim milk for 1 h, incubated with primary antibodies (4 °C overnight), washed with TBST, incubated with HRP-conjugated secondary antibodies (room temperature, 1 h), and visualized using ECL substrate (Bio-Rad).

#### 4.2.8. SIRT1 Plasmid Transfection

The SIRT1-overexpressing plasmid (SIRT1-OE, stored at −80 °C) was thawed. For transfection, 1.5 μL of endotoxin-free SIRT1-OE plasmid (1 μg/μL) was mixed with 125 μL Opti-MEM™ (Gibco, Grand Island, NY, USA). Separately, 5 μL Lipofectamine 3000 (Invitrogen, Carlsbad, CA, USA) was diluted in 125 μL Opti-MEM™, incubated for 5 min, and combined with the plasmid mixture. After 5 min of incubation, the mixture was added to the HepG2, PLC/PRF/5, and MHCC97H cells (logarithmic phase) for 6 h. The medium was replaced with fresh DMEM, and cells were cultured for 24 h before subsequent experiments.

#### 4.2.9. Generation of SIRT1-Stably Overexpressing HepG2 Cells

HepG2 cells in the logarithmic phase were seeded into 6-well plates. At 50–60% confluency, the medium was replaced with lentiviral transduction solution (prepared per the manufacturer’s protocol, Lenti-X (Kusatsu, Japan), Takara (Kusatsu, Japan)) and incubated for 16 h. After replacing with fresh DMEM for 72 h, fluorescence was observed under a fluorescence microscope (Olympus, Shinjuku City, Japan). Successfully transduced cells were selected with 2 μg/mL puromycin (Sigma-Aldrich) for 48 h, then seeded into 96-well plates at 1 cell/well in puromycin-containing medium. Monoclonal colonies were expanded, and SIRT1 overexpression was confirmed by qPCR and Western blot. Stable cell lines were cryopreserved in liquid nitrogen.

### 4.3. Xenograft Tumor Model in Nude Mice

#### 4.3.1. Cell Preparation

HepG2 cells and SIRT1-overexpressing HepG2 cells in the logarithmic growth phase were harvested, washed twice with PBS, digested, and centrifuged. Cell density was adjusted to 1 × 10^7^ cells/mL in a mixture of serum-free DMEM and Matrigel (3:7 ratio), kept on ice. The cell suspension was subcutaneously inoculated into the flanks of nude mice.

#### 4.3.2. Drug Administration and Specimen Collection

After tumor formation, **1-P** (dissolved in saline with 0.01% DMSO) was administered via tail vein injection. Mice were randomly divided into three groups: control, experimental group 1 (1 mg/kg), and experimental group 2 (2 mg/kg). Treatment was administered every other day. Mouse activity and tumor growth were monitored. Tumor dimensions were measured using a vernier caliper, and body weight was recorded with an electronic balance. Tumor volume was calculated as *L* × *W*^2^/2, where *L* is the longest diameter and *W* is the shortest diameter. After treatment, mice were euthanized, and tumors were excised, weighed, and measured. Heart, liver, spleen, lungs, and kidneys were collected for further analysis. Experiments of xenograft tumor model in nude mice were approved and met the ethical standards of the Institutional Review Board of the Laboratory Animal Ethics Committee, Guangdong Medical University (approval number: GDMU-2025-000027).

#### 4.3.3. Statistical Analysis

Data were analyzed using SPSS 26.0 (IBM) and GraphPad Prism 9.0. Continuous variables are expressed as mean ± standard deviation (SD). Homogeneity of variance was assessed: for homogeneous data, an independent samples *t*-test (two groups) or one-way ANOVA (multiple groups) was applied; for heterogeneous data, the Kruskal–Wallis test was used. *p*-value < 0.05 was considered statistically significant. All experiments were independently repeated three times.

## 5. Conclusions

In summary, 5,10,15-triethoxycarbonyl P(V) corrole can inhibit the viability of HepG2, PLC/PRF5, and MHCC97H hepatocellular carcinoma cells, clone and multiply, increase the level of mitochondrial membrane potential, promote the increase of intracellular ROS level, and induce the apoptosis of hepatoma cells. The expression of SIRT protein affects the drug activity of 5,10,15-triethoxycarbonyl P(V) corrole to some extent. SIRT1 protein on the surface may be an important protein in the photodynamic therapy of 5,10,15-triethoxycarbonyl P(V) corrole. It provides a new idea for the future study of the molecular mechanism of corrole.

## Data Availability

All data generated or analyzed in this study are included in this publication.

## Supplementary Materials

The following supporting information can be downloaded at: https://www.mdpi.com/article/10.3390/ph18081226/s1. Figure S1: Effects of **1-P** treatment on cell viability in hepatocellular carcinoma cells. Figure S2: **1-P** induces mitochondrial depolarization and ROS accumulation in hepatocellular carcinoma cells. Figure S3: SIRT1 overexpression modulates **1-P**-mediated cytotoxic and anti-proliferative effects in hepatocellular carcinoma cells. Figure S4: SIRT1 overexpression preserves mitochondrial membrane potential and reduces ROS accumulation in **1-P**-treated hepatocellular carcinoma cells. Figure S5: Stable SIRT1-overexpressing HepG2 cell line established via lentiviral transduction. Figure S6: **1-P** exhibits a favorable safety profile in tumor-bearing mice.
