# Supplementary material for: SIRT1 Modulates the Photodynamic Anticancer Activity of 5,10,15-Triethoxycarbonyl P(V) Corrole in Hepatocellular Carcinoma
Source: Pharmaceuticals (Basel). 2025 Aug 20;18(8):1226. doi: 10.3390/ph18081226 (PMC12388956; doi:10.3390/ph18081226)
Supplement: Supplementary file 1 [file pharmaceuticals-18-01226-s001.zip › pharmaceuticals-3778296-supplementary.pdf]

Supplementary Information

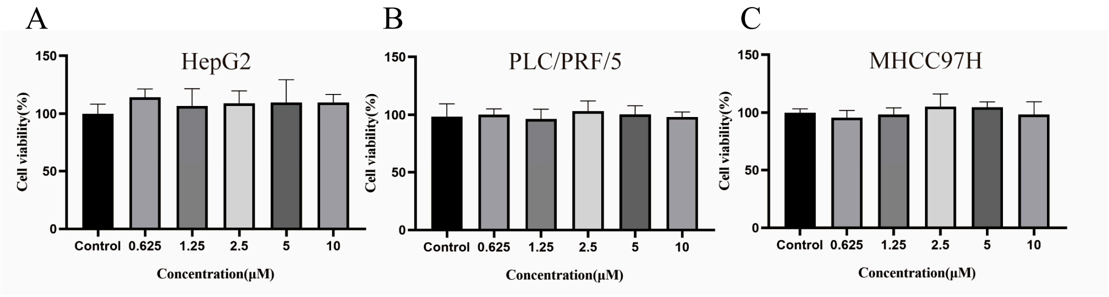

Figure S1. Effects of **1-P** treatment on cell viability in hepatocellular carcinoma cells. (A) HepG2, (B) PLC/PRF/5 and (C) MHCC97H cells were treated with varying concentrations of **1-P** (10, 5, 2.5, 1.25, 0.625 μM) for 4 h, followed by 24 h incubation in the dark.

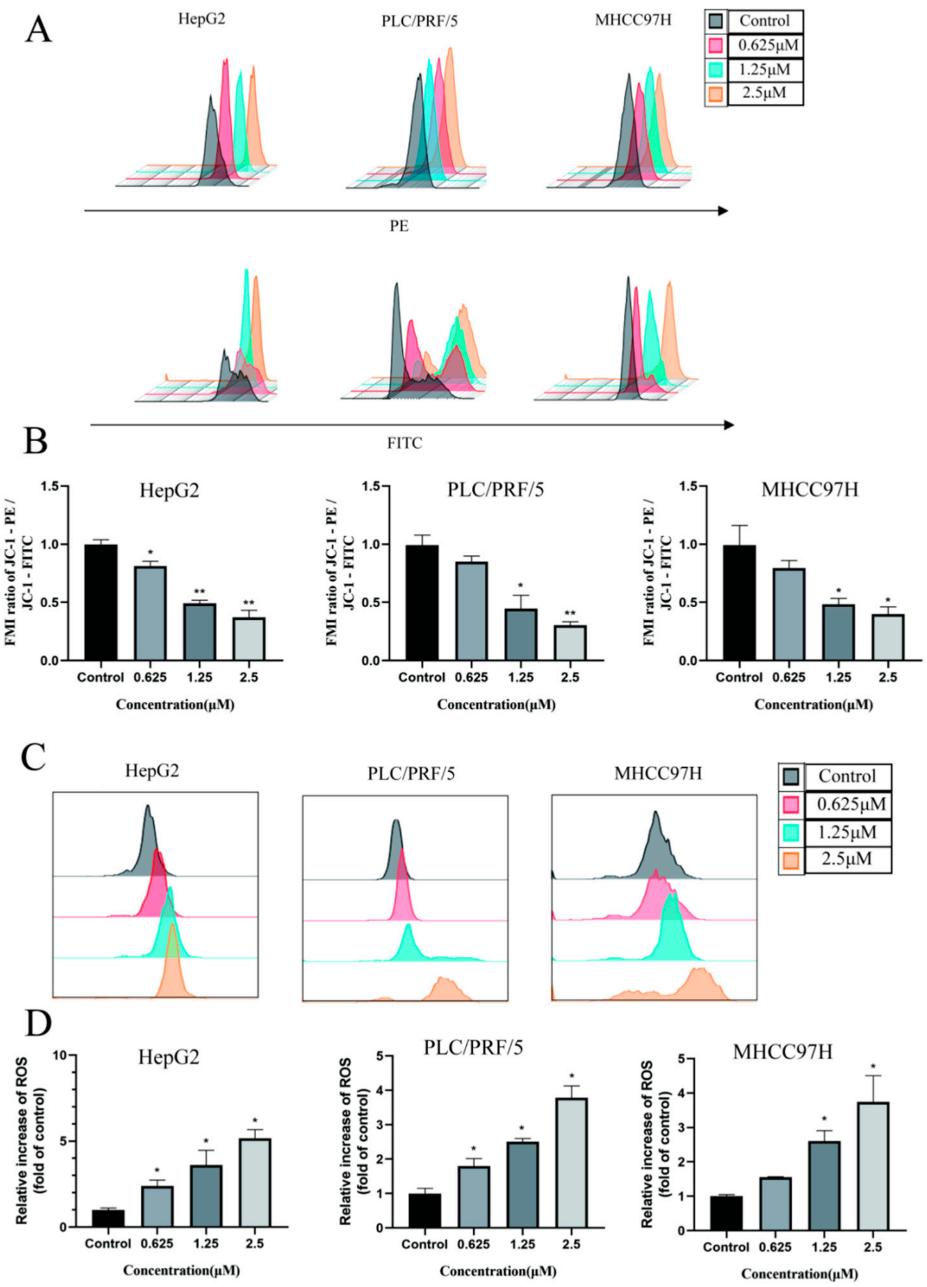

Figure S2. 1-P induces mitochondrial depolarization and ROS accumulation in hepatocellular carcinoma cells. (A) JC-1 staining (red/green fluorescence) reflecting mitochondrial membrane potential (ΔΨm) in HCC cells treated with **1-P** (0.625, 1.25, 2.5 μM) and 625 nm irradiation. (B) Quantitative analysis of JC-1 red/green fluorescence ratio (PE/FITC), indicating ΔΨm collapse (*$p < 0.05$, **$p < 0.01$ vs. control). (C) DCFH-DA fluorescence imaging showing intracellular ROS levels in **1-P**-treated HCC cells. (D) ROS fluorescence intensity quantification (fold change vs. control, mean ± SD; *$p < 0.05$, **$p < 0.01$).

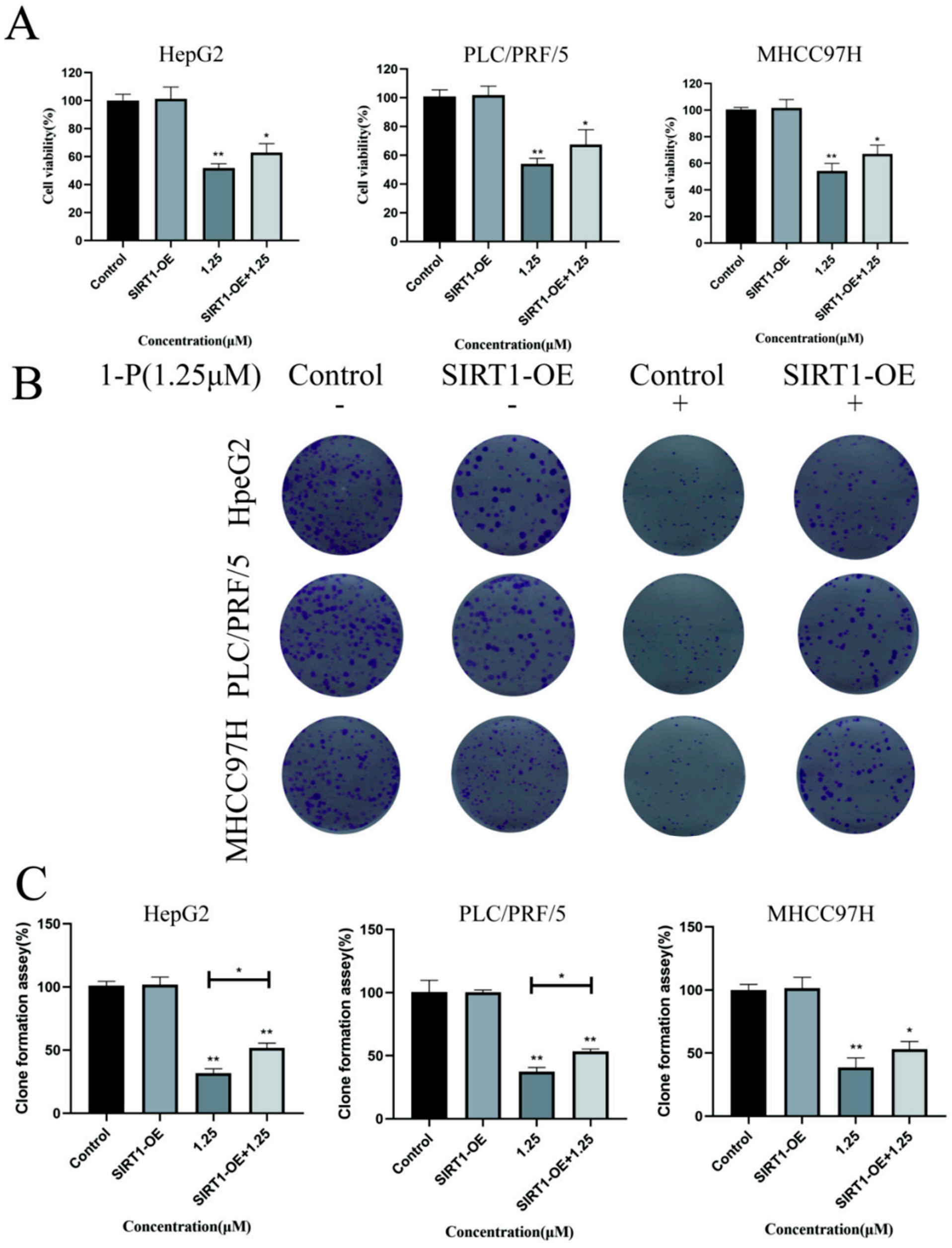

Figure S3. SIRT1 overexpression modulates **1-P**-mediated cytotoxic and anti-proliferative effects in hepatocellular carcinoma cells. (A) Cell viability assessed by MTT assay after transient transfection with SIRT1-overexpressing plasmids (SIRT1-OE), 1.25 μM **1-P** treatment (4 h with 625 nm light irradiation), and 20 h incubation. (B) and (C) Clonogenic survival analysis under identical treatment conditions. Data represent mean ± SD (*p < 0.05, **p < 0.01 vs. control group).

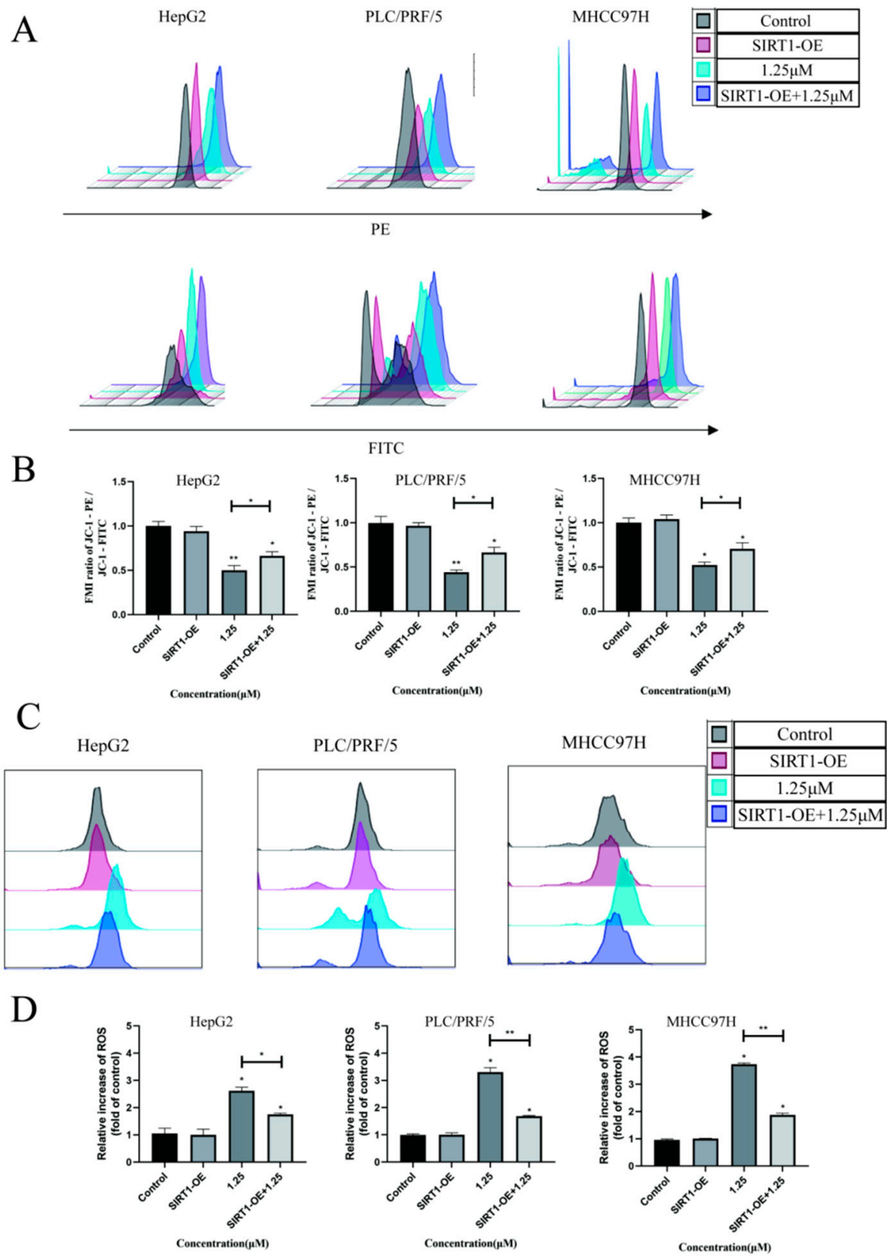

Figure S4. SIRT1 overexpression preserves mitochondrial membrane potential and reduces ROS accumulation in **1-P**-treated hepatocellular carcinoma cells. (A) JC-1 staining (red/green fluorescence) showing mitochondrial membrane potential (ΔΨm) changes in HCC cells transiently transfected with SIRT1-overexpressing plasmids (SIRT1-OE) and treated with 1.25 μM **1-P** (4 h + 625 nm light irradiation). (B) Quantitative analysis of JC-1 red/green fluorescence ratio. (C) DCFH-DA fluorescence imaging of intracellular ROS levels under identical treatment conditions. (D) ROS fluorescence intensity quantification (mean ± SD; *p < 0.05, **p < 0.01 vs. control group).

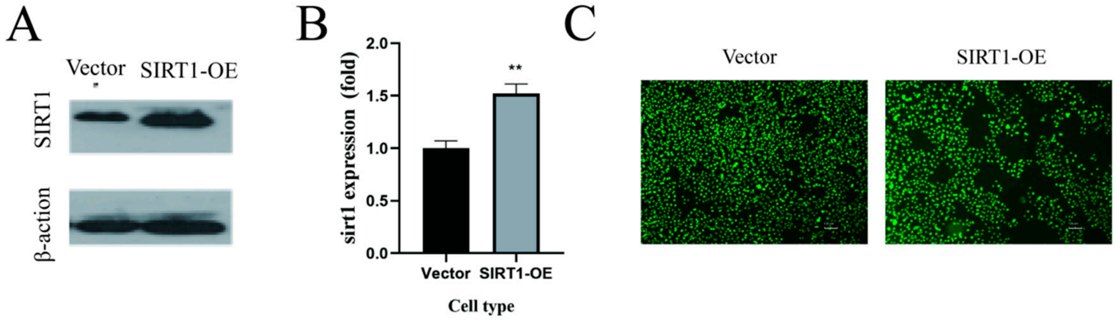

Figure S5. Stable SIRT1-overexpressing HepG2 cell line established via lentiviral transduction. (A) Western blot analysis confirming sustained SIRT1 protein overexpression in stably transduced cells (SIRT1-OE) compared to empty vector controls (Vector). (B) qPCR quantification demonstrating 1.5-fold higher SIRT1 mRNA levels in SIRT1-OE cells (****p* < 0.001 vs. Vector). (C) Fluorescence microscopy images (20×) showing GFP-positive populations in stable SIRT1-OE cells (right) versus minimal background signal in vector controls (left).

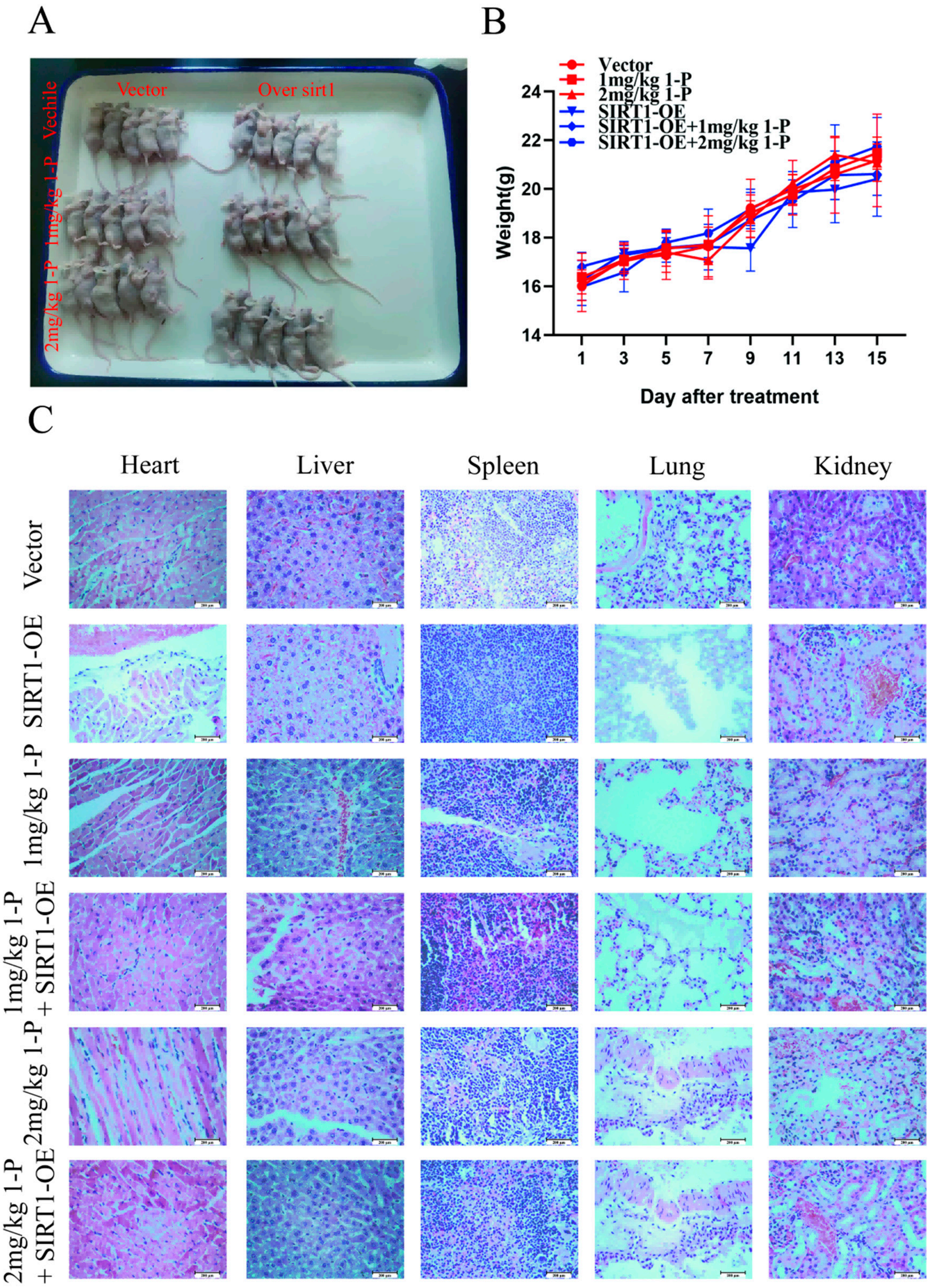

Figure S6. 1-P exhibits favorable safety profile in tumor-bearing mice. (A) Schematic of experimental timeline and subcutaneous tumor implantation. (B) Body weight dynamics of mice during **1-P** treatment (mean ± SD, n = 6/group). (C) Representative H&E-stained sections (200×) of heart, liver, spleen, lung, and kidney tissues from control and **1-P**-treated groups, showing no histopathological abnormalities.
